# Comparison of quantitative real-time PCR and direct immunofluorescence for the detection of *Pneumocystis jirovecii*

**DOI:** 10.1371/journal.pone.0180589

**Published:** 2017-07-06

**Authors:** Bhavani Moodley, Stefano Tempia, John Andrew Frean

**Affiliations:** 1 Centre for Emerging, Zoonotic and Parasitic Diseases, National Institute for Communicable Diseases of the National Health Laboratory Service, Johannesburg, South Africa; 2 Influenza Division, Centers for Disease Control and Prevention, Atlanta, Georgia, United States of America; 3 Influenza Program, Centers for Disease Control and Prevention, Pretoria, South Africa; 4 Centre for Respiratory Diseases and Meningitis, National Institute for Communicable Diseases of the National Health Laboratory Service, Johannesburg, South Africa; 5 Wits Research Institute for Malaria, University of the Witwatersrand, Johannesburg, South Africa; Stony Brook University, UNITED STATES

## Abstract

**Background:**

*Pneumocystis* pneumonia (PCP) is a serious risk for HIV-positive patients. Asymptomatic infection or colonisation with *P*. *jirovecii* has been shown to occur frequently. PCR assays frequently identify such cases, due to their high sensitivity. Quantitative real-time PCR (qPCR) gene copy number cut-off values have been suggested to differentiate colonisation and infection; these need to be standardised for routine use. We compared the results of qPCR with an immunofluorescence assay (IFA) to determine a specific cut-off value.

**Methods:**

From March 2005 through June 2009, induced sputum specimens were collected from adult patients who were clinically suspected of having PCP, at the Chris Hani Baragwanath Hospital in Gauteng, South Africa. Laboratory diagnosis of PCP was done by a conventional direct IFA and a qPCR assay. A receiver operating characteristic (ROC) analysis was performed to determine a suitable copy number cut-off value.

**Results:**

*P*. *jirovecii* was identified in 51% (156/305) and 67% (204/305) of specimens using IFA and qPCR, respectively. The cut-off value for the qPCR that best predicted the IFA results was 78 copies/5 μl (area under ROC curve 0.92). The sensitivity and specificity of qPCR using this cut-off was 94.6% and 89.1%, respectively, compared with the IFA.

**Discussion:**

The results of the ROC curve analysis indicate an excellent predictive value of the qPCR using the proposed cut-off. However, the IFA test is an imperfect gold standard and so this cut-off should not be used in isolation; clinical data should also contribute to the interpretation of the qPCR result.

## Introduction

*Pneumocystis* pneumonia (PCP) is a frequent opportunistic infection in HIV-positive patients but also affects other immunocompromised patients [1,2]. Healthy individuals have been shown to harbor the organism without clinical signs of infection [1,3,4]. These findings suggest that asymptomatic infection or colonisation occurs frequently, and may play a large role in the transmission of PCP.

With the rapid advancements in molecular biology, an increasing number of laboratories are adopting polymerase chain reaction (PCR) as an option for diagnostic testing. However, resource-limited laboratories continue to use conventional methods, for example immunofluorescence assays (IFA) are still commonly used for the diagnosis of *P*. *jirovecii* [5]. PCR, especially quantitative real-time PCR (qPCR), is a highly sensitive assay compared to most conventional methods. Nonetheless, this increased sensitivity may result in the identification of cases of colonisation in addition to infection, making results less valuable [6]. DNA copy number cut-off values to differentiate colonisation and infection for qPCR assays have been suggested but need to be standardised in local settings for routine use [7–10].

Using data from a PCP study carried out on HIV-infected adults hospitalised with pneumonia, we compared two *P*. *jirovecii* diagnostic methods, namely, IFA and qPCR. Furthermore, we used the qPCR assay to determine a copy number cut-off, with the IFA as the ‘gold standard’ in our study population.

## Materials and methods

### Patients’ enrolment and sample collection procedures

The study was done under ethical approval (clearance number M040612) of the Human Research Ethics Committee (Medical) of the University of the Witwatersrand, Johannesburg, South Africa. Written informed consent was obtained from subjects admitted with a diagnosis of pneumonia to the Chris Hani Baragwanath Hospital in Soweto, South Africa. An experienced pulmonologist evaluated patients clinically and radiologically as part of routine care during 2005–2009. Adult patients (≥18 years of age) suspected of having PCP were enrolled, and following nebulised sterile saline inhalation under a trained nurse’s supervision, induced sputum samples were collected. Specimens were refrigerated at 4°C and sent on icepacks to the National Institute for Communicable Diseases for laboratory investigations, which were done within 24 hours of sample receipt.

### Immunofluorescence assay

Induced sputum specimens were pre-digested with 1.4-dithiothreitol (DTT, Roche Diagnostics GmbH, Mannheim, Germany) and then processed for immunofluorescence using the Light Diagnostics Direct Immunofluorescence Assay (IFA) kit for the detection of *P*. *jirovecii* (Millipore Corporation, Billerica, MA 01821) according to manufacturer’s instructions. Slides were examined with an ultraviolet light-equipped microscope under 400× magnification for any fluorescing antibody-antigen complexes. If two or more typical ‘honeycomb’ clumps of *P*. *jirovecii* cysts were seen, the specimen was designated ‘positive’ and categorised subjectively as weak positive (+), positive (++), or strong positive (+++). If only one confirmed or suspicious clump and/or cyst was seen, the result was recorded as ‘possibly positive’. Fluorescing particles that were markedly smaller than a typical cyst were ignored.

### Quantitative real-time PCR

Induced sputa were washed with phosphate-buffered saline (Diagnostic Media Products, Johannesburg, South Africa) to remove DTT from the sample. Samples were digested with bacterial lysis buffer (Roche Diagnostics) and proteinase K (Roche Diagnostics) for 2 hours at 56°C. DNA was extracted using the Roche MagNA Pure Compact (Roche Diagnostics) and the MagNA Pure Compact Nucleic Acid isolation kit (Roche Diagnostics), as per the manufacturer’s instructions for the bacterial lysis protocol. Extracted DNA (100 μl/sample) was frozen at -70°C. Fungal load was determined with a qPCR assay targeting a gene region coding for the *P*. *jirovecii* mitochondrial large subunit (MtLSU) rRNA, as previously described [11], using a Life Technologies 7500 Real-time PCR Instrument (Life Technologies, Foster City, California). Oligonucleotide primers and probe were commercially synthesised; forward primer LSU1 (5´-AAA TAA ATA ATC AGA CTA TGT GCG ATA AGG-3´), reverse primer LSU2 (5´-GGG AGC TTT AAT TAC TGT TCT GGG-3´) and probe LSUP1 (FAM 5´-AGA TAG TCC AAA GGG AAA C-3´TAMRA). Result analysis was done with the Life Technologies 7500 System Software. Real-time PCR results were extrapolated from a standard curve and were expressed as target sequence copy number per 5 μl of extracted DNA. This is further referred to as the ‘fungal load’ and correlates with the qPCR cycle threshold (Ct) values. A fungal load greater than zero was taken as a positive DNA result. Samples that were IFA-positive and qPCR-negative were tested for PCR inhibition using an RNaseP PCR, as previously described [12]. Samples were tested once each by IFA and qPCR; positive and negative controls were used for both assays.

### Statistical analysis

To determine the statistical measures of performance of the qPCR assay, the IFA-possibly positive results were combined with the IFA-positive results. We used a receiver operating characteristic (ROC) analysis to determine the optimal fungal load/Ct value cut-off of the qPCR in relation to the IFA results (considered as the gold standard in this study). We then estimated the sensitivity, specificity, positive and negative predictive value of the assay using the identified cut-off value. The performance of the identified cut-off value was assessed by calculating the area under the ROC curve.

## Results

### Patients

During the study period, 266 adult patients with clinically-suspected PCP were recruited. The median patient age was 34 years, and the ratio of males to females was 1:1.3. All patients were either laboratory confirmed as, or highly suspected on clinical grounds to be, HIV-positive. A total of 305 respiratory specimens was collected for testing; some patients had more than one sample collected during their hospital stay.

### Immunofluorescence assay

**Fig 1:**
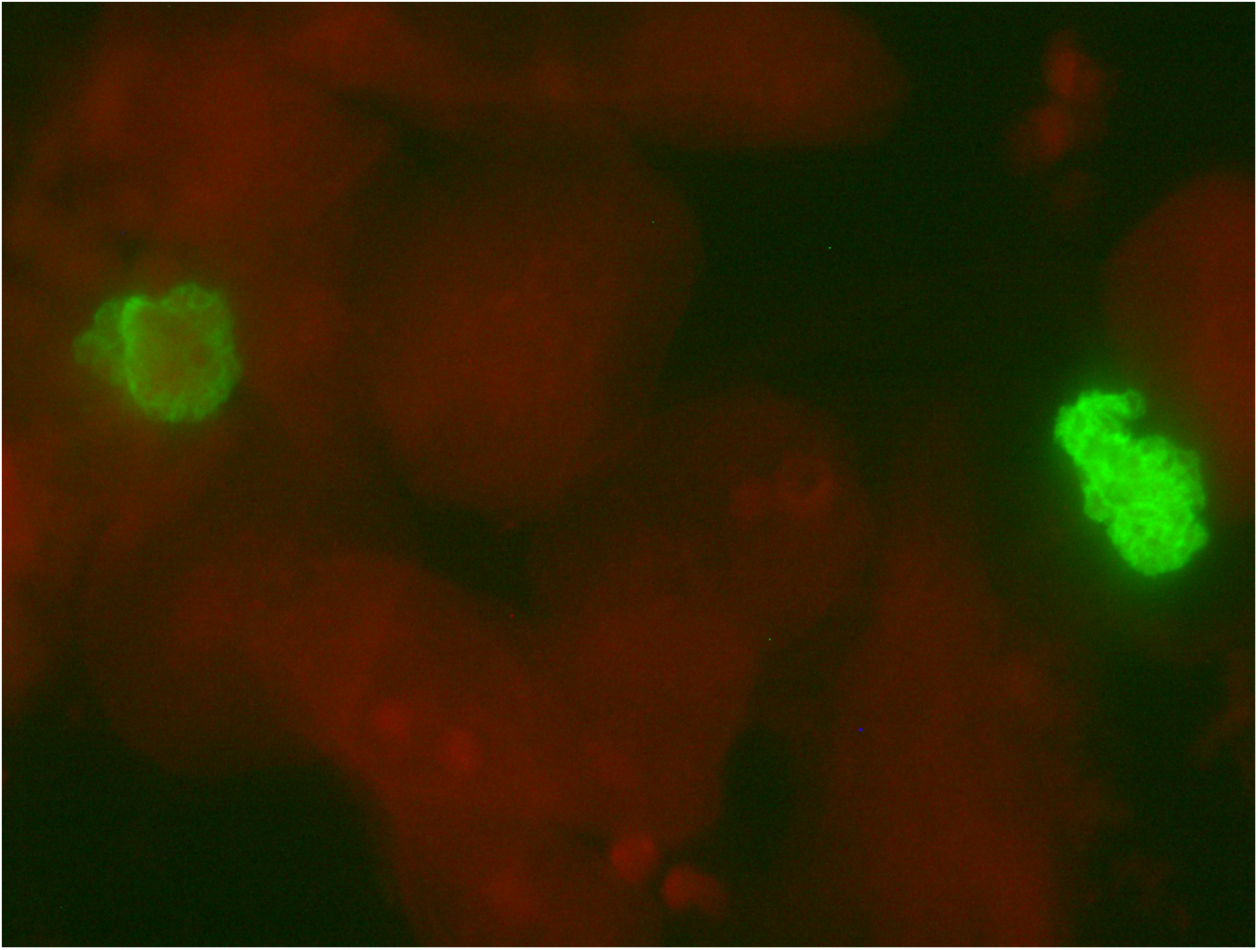
Micrograph showing two fluorescing green clusters of *P*. *jirovecii* cysts on a red-stained background, 400x magnification.

Of the 305 specimens tested for *P*. *jirovecii* by the IFA detection method, 51% (156/305) were IFA positive, 4% (11/305) were IFA possibly positive and 45% (138/305) were IFA negative. Fig 1 shows two fluorescing clusters of *P*. *jirovecii* cysts.

### Quantitative real-time PCR

Quantitative real-time PCR was performed on DNA extracts from all 305 specimens. Sixty-seven percent (204/305) of the specimens were positive for *P*. *jirovecii* DNA (i.e. they had a fungal load greater than 0) (Table 1).

**Table 1:** Comparison of the *P*. *jirovecii* immunofluorescence assay (IFA) and the quantitative real-time PCR (qPCR) assay results, Soweto, South Africa, 2005–2009 (N = 305).

		IFA Result			
		Positive	Possibly positive	Negative	Total
**qPCR Result**	**Positive**	153	10	41	204
	**Negative**	3	1	97	101
	**Total**	156	11	138	305

Using the IFA test as the gold standard, the sensitivity and specificity of the qPCR assay were calculated to be 98.2% and 70.7%, respectively. The positive and negative predictive values were 80.1% and 96.5%, respectively. There were four false negative qPCR results; these were tested for PCR inhibition and the IFA slides were re-examined. Two of these specimens were shown to contain PCR inhibitors, using the RNase P PCR assay. One specimen was regarded as having an incorrect positive IFA result, as it did not correspond with the typical morphology of *P*. *jirovecii* on slide review.

The median fungal load correlated well with the IFA results (Table 2).

**Table 2:** qPCR fungal load of *P*. *jirovecii* DNA for each immunofluorescence assay (IFA) result category, Soweto, South Africa, 2005–2009.

IFA result category	qPCR assay results expressed as *P*. *jirovecii* fungal load (copy number per 5 μl) Median (range)
IFA-negative	**0** (0–147 500)
IFA-possibly positive	**401** (0–2 566)
IFA-positive	**12 270** (7–704 257)
• IFA +	• **195** (7–48 923)
• IFA ++	• **1 779** (93–56 260)
• IFA +++	• **23 643** (137–704 257)

The ROC analysis indicated that, in comparison with the IFA results, the optimal cut-off value for the qPCR was 78 DNA copies or a Ct value of 38.19, which correctly classified 92.1% of the IFA results. The area under the ROC curve was 0.92 (95% confidence interval: 0.88–0.94), which indicates an excellent predictive value of PCR using this cut-off value. The sensitivity and specificity of the qPCR using this cut-off were 94.6% and 89.1%, respectively.

## Discussion

In this study, we compared the IFA method to qPCR for the detection of *P*. *jirovecii*, in a South African cohort of immunocompromised patients. This is the first study in South Africa to use ROC analysis to compare IFA with qPCR. The former method identified 51% of specimens as positive, and the latter 67%. The IFA staining method allows for the *Pneumocystis* organisms to be microscopically identified, whereas the qPCR amplifies target DNA sequences. Therefore it must be noted that DNA in a specimen could be from dead organisms and therefore may not be clinically relevant [13].

Using the IFA as the gold standard, the qPCR had 41 false-positive and four false-negative results. The false-positive results were mostly in specimens with low fungal loads (median copy number, 45) that were not detected by the IFA. As these specimens had overall lower median copy numbers of the target gene sequences, compared to the true-positive results, they may have originated from patients who were colonised rather than infected with *P*. *jirovecii* [13]. If this is true, then these patients probably had other respiratory diseases to account for their clinical presentations on enrolment. In the USA, a large proportion (69%) of HIV-positive patients without PCP, were found to be colonised with *P*. *jirovecii* using PCR [14].

The four qPCR false-negative results were further investigated. Two specimens contained PCR inhibitors, one was deemed a false-positive IFA result (reader error) on slide review and the last one was assumed to be due to unidentified technical error. The IFA method has been shown to have the highest sensitivity but the lowest specificity when compared to three other staining methods [5]. Non-specific staining is a limitation of IFA kits for *P*. *jirovecii* [15], especially if coupled with inexperienced microscopists. The strict use of defined morphological criteria, as we did, should improve the specificity of IFA. Khan *et al*. compared PCR and IFA assays and concluded that immunofluorescence should not be used solely for the diagnosis of PCP [13]. They further stated that PCR should be used when the IFA result is negative and the patient is suspected of having PCP.

A major problem when evaluating tests for PCP diagnosis is the lack of a true gold standard. This is mainly due to the non-existence of a culture system for *P*. *jirovecii*. The IFA test was chosen as the gold standard in this study because it was the current routine diagnostic test for PCP used by the laboratory. The use of clinical and radiological criteria may serve as a good gold standard for *P*. *jirovecii* diagnosis, as was used by Fujisawa *et al*. [16]; however, radiological findings were not available for analysis in this study.

Our results showed a good correlation between the qPCR median fungal load and IFA result i.e. low copy number for IFA-negative specimens and increasingly higher copy numbers for IFA-possibly positive and IFA-positive specimens. The median copy number was used instead of the mean to reduce the effect of outliers. However, there was a large overlap in the range of copy numbers in each category. The optimal cut-off value (78 DNA copies/5 μl or Ct value of 38.19) that was derived from the ROC analysis indicated an excellent predictive value of this PCR assay when compared against the IFA results. As previously discussed, the IFA test is not a completely reliable gold standard because it does not always distinguish true infection from colonisation; therefore this copy number cut-off is subject to this limitation.

There are many publications that have suggested qPCR cut-off values for *Pneumocystis* infection and colonisation, including some that incorporate a ‘grey zone’ for indeterminate results [7,9,10,17–19]. However, these cut-offs cannot be applied to different clinical situations and different assays [18,20]. When using previously established cut-offs it must be remembered that fungal loads will vary according to study parameters, including study population, specimen type and quality, and test method used. It has been shown that HIV-infected PCP patients have higher fungal loads than HIV-negative patients [19–21]. Furthermore paediatric patients may have different loads compared to adults. Specimen type and quality influences test results; for PCP, bronchoalveolar lavage and induced sputum samples are accepted to be the preferred types [1]. A cut-off value established for these sample types cannot be used for studies done on less-optimal specimen types, such as nasopharyngeal aspirates. Cut-off values will also vary based on the test methods used; PCR assays have various gene targets, sensitivities and units of measurement. Therefore from our results, a cut-off value of 78 DNA copies/5 μl (or a Ct value of 38.19) can be used to differentiate cases of *P*. *jirovecii* infection and colonisation when testing induced sputum specimens from HIV-positive patients, using the specified qPCR assay, in our setting.

It has been suggested that qPCR cut-offs be combined with another test such as IFA or β-1,3-D-glucan to create a diagnostic algorithm [22,23]. This is especially advisable for routine diagnostic testing.

In conclusion, while qPCR cut-offs are useful, they cannot be universally applied but should rather be established using a pilot study with the same study parameters. However, as with many laboratory tests, irrespective of the method used to diagnose PCP, clinical interpretation, in light of patient presentation, is the ultimate deciding factor for patient treatment.

### Disclaimer

The findings and conclusions in this report are those of the authors and do not necessarily represent the official position of the US Centers for Disease Control and Prevention, USA or the National Institute for Communicable Diseases, South Africa.

## Supporting information

S1 File Excel file showing data and receiver operator characteristic analysis. (XLS)

## References

[1] Thomas CF Jr., Limper AH. Pneumocystis pneumonia. N Engl J Med 2004 6 10;350(24):2487–98. doi: 10.1056/NEJMra032588 15190141 10.1056/NEJMra032588

[2] Thomas CF Jr., Limper AH. Current insights into the biology and pathogenesis of *Pneumocystis* pneumonia. Nat Rev Microbiol 2007 4;5(4):298–308. doi: 10.1038/nrmicro1621 17363968 10.1038/nrmicro1621

[3] Davis JL, Welsh DA, Beard CB, Jones JL, Lawrence GG, Fox MR, et al *Pneumocystis* colonisation is common among hospitalised HIV infected patients with non-*Pneumocystis* pneumonia. Thorax 2008 4;63(4):329–34. doi: 10.1136/thx.2007.088104 18024536 10.1136/thx.2007.088104

[4] Medrano FJ, Montes-Cano M, Conde M, de la HC, Respaldiza N, Gasch A, et al *Pneumocystis jirovecii* in general population. Emerg Infect Dis 2005 2;11(2):245–50. doi: 10.3201/eid1102.040487 15752442 10.3201/eid1102.040487 PMC3320436

[5] Procop GW, Haddad S, Quinn J, Wilson ML, Henshaw NG, Reller LB, et al Detection of *Pneumocystis jiroveci* in respiratory specimens by four staining methods. J Clin Microbiol 2004 7;42(7):3333–5. doi: 10.1128/JCM.42.7.3333-3335.2004 15243109 10.1128/JCM.42.7.3333-3335.2004 PMC446244

[6] Sing A, Trebesius K, Roggenkamp A, Russmann H, Tybus K, Pfaff F, et al Evaluation of diagnostic value and epidemiological implications of PCR for *Pneumocystis carinii* in different immunosuppressed and immunocompetent patient groups. J Clin Microbiol 2000 4;38(4):1461–7. 10747126 10.1128/jcm.38.4.1461-1467.2000 PMC86465

[7] Alanio A, Desoubeaux G, Sarfati C, Hamane S, Bergeron A, Azoulay E, et al Real-time PCR assay-based strategy for differentiation between active *Pneumocystis jirovecii* pneumonia and colonization in immunocompromised patients. Clin Microbiol Infect 2010 10 14;10–0691. 10.1111/j.1469-0691.2010.03400.x 20946413

[8] Helweg-Larsen J. S-adenosylmethionine in plasma to test for *Pneumocystis carinii* pneumonia. Lancet 2003 4 12;361(9365):1237 doi: 10.1016/S0140-6736(03)13027-9 12699946 10.1016/S0140-6736(03)13027-9

[9] Huggett JF, Taylor MS, Kocjan G, Evans HE, Morris-Jones S, Gant V, et al Development and evaluation of a real-time PCR assay for detection of *Pneumocystis jirovecii* DNA in bronchoalveolar lavage fluid of HIV-infected patients. Thorax 2008 2;63(2):154–9. doi: 10.1136/thx.2007.081687 17693588 10.1136/thx.2007.081687

[10] Fillaux J, Malvy S, Alvarez M, Fabre R, Cassaing S, Marchou B, et al Accuracy of a routine real-time PCR assay for the diagnosis of *Pneumocystis jirovecii* pneumonia. J Microbiol Methods 2008 10;75(2):258–61. doi: 10.1016/j.mimet.2008.06.009 18606198 10.1016/j.mimet.2008.06.009

[11] Dini L, Du PM, Frean J, Fernandez V. High prevalence of dihydropteroate synthase mutations in *Pneumocystis jirovecii* isolated from patients with *Pneumocystis* pneumonia in South Africa. J Clin Microbiol 2010 6;48(6):2016–21. doi: 10.1128/JCM.02004-09 20351205 10.1128/JCM.02004-09 PMC2884465

[12] Carvalho MG, Tondella ML, McCaustland K, Weidlich L, McGee L, Mayer LW, et al Evaluation and improvement of real-time PCR assays targeting *lytA*, *ply*, and *psaA* genes for detection of pneumococcal DNA. J Clin Microbiol 2007 8;45(8):2460–6. doi: 10.1128/JCM.02498-06 17537936 10.1128/JCM.02498-06 PMC1951257

[13] Khan MA, Farrag N, Butcher P. Diagnosis of *Pneumocystis carinii* pneumonia: immunofluorescence staining, simple PCR or nPCR. J Infect 1999 7;39(1):77–80. 10468133 10.1016/s0163-4453(99)90106-8

[14] Huang L, Crothers K, Morris A, Groner G, Fox M, Turner JR, et al *Pneumocystis* colonization in HIV-infected patients. J Eukaryot Microbiol 2003;50 Suppl:616–7:616–7. 14736184 10.1111/j.1550-7408.2003.tb00651.x

[15] Robberts FJ, Liebowitz LD, Chalkley LJ. Polymerase chain reaction detection of *Pneumocystis jiroveci*: evaluation of 9 assays. Diagn Microbiol Infect Dis 2007 8;58(4):385–92. doi: 10.1016/j.diagmicrobio.2007.02.014 17689766 10.1016/j.diagmicrobio.2007.02.014

[16] Fujisawa T, Suda T, Matsuda H, Inui N, Nakamura Y, Sato J, et al Real-time PCR is more specific than conventional PCR for induced sputum diagnosis of *Pneumocystis* pneumonia in immunocompromised patients without HIV infection. Respirology 2009 3;14(2):203–9. doi: 10.1111/j.1440-1843.2008.01457.x 19210645 10.1111/j.1440-1843.2008.01457.x

[17] Larsen HH, Masur H, Kovacs JA, Gill VJ, Silcott VA, Kogulan P, et al Development and evaluation of a quantitative, touch-down, real-time PCR assay for diagnosing *Pneumocystis carinii* pneumonia. J Clin Microbiol 2002 2;40(2):490–4. doi: 10.1128/JCM.40.2.490-494.2002 11825961 10.1128/JCM.40.2.490-494.2002 PMC153364

[18] Maillet M, Maubon D, Brion JP, Francois P, Molina L, Stahl JP, et al *Pneumocystis jirovecii* (Pj) quantitative PCR to differentiate Pj pneumonia from Pj colonization in immunocompromised patients. Eur J Clin Microbiol Infect Dis 2014 3;33(3):331–6. doi: 10.1007/s10096-013-1960-3 23990137 10.1007/s10096-013-1960-3 PMC7101903

[19] Fauchier T, Hasseine L, Gari-Toussaint M, Casanova V, Marty PM, Pomares C. Detection of *Pneumocystis jirovecii* by quantitative PCR to differentiate colonization and pneumonia in immunocompromised HIV-positive and HIV-negative patients. J Clin Microbiol 2016 6;54(6):1487–95. doi: 10.1128/JCM.03174-15 27008872 10.1128/JCM.03174-15 PMC4879311

[20] Calderon EJ, Gutierrez-Rivero S, Durand-Joly I, Dei-Cas E. *Pneumocystis* infection in humans: diagnosis and treatment. Expert Rev Anti Infect Ther 2010 6;8(6):683–701. doi: 10.1586/eri.10.42 20521896 10.1586/eri.10.42

[21] Takahashi T, Goto M, Endo T, Nakamura T, Yusa N, Sato N, et al *Pneumocystis carinii* carriage in immunocompromised patients with and without human immunodeficiency virus infection. J Med Microbiol 2002 7;51(7):611–4. doi: 10.1099/0022-1317-51-7-611 12132780 10.1099/0022-1317-51-7-611

[22] Muhlethaler K, Bogli-Stuber K, Wasmer S, von GC, Dumont P, Rauch A, et al Quantitative PCR to diagnose *Pneumocystis* pneumonia in immunocompromised non-HIV patients. Eur Respir J 2012 4;39(4):971–8. doi: 10.1183/09031936.00095811 21920890 10.1183/09031936.00095811

[23] Robert-Gangneux F, Belaz S, Revest M, Tattevin P, Jouneau S, Decaux O, et al Diagnosis of *Pneumocystis jirovecii* pneumonia in immunocompromised patients by real-time PCR: a 4-year prospective study. J Clin Microbiol 2014 9;52(9):3370–6. doi: 10.1128/JCM.01480-14 25009050 10.1128/JCM.01480-14 PMC4313164

